# Comparative analyses of longevity and senescence reveal variable survival benefits of living in zoos across mammals

**DOI:** 10.1038/srep36361

**Published:** 2016-11-07

**Authors:** Morgane Tidière, Jean-Michel Gaillard, Vérane Berger, Dennis W. H. Müller, Laurie Bingaman Lackey, Olivier Gimenez, Marcus Clauss, Jean-François Lemaître

**Affiliations:** 1 Université de Lyon, F-69000, Lyon; Université Lyon 1; CNRS, UMR5558, Laboratoire de Biométrie et Biologie Evolutive, F-69622, Villeurbanne, France; 2 Zoologischer Garten Halle GmbH, Fasanenstr. 5a, 06114 Halle (Saale), Germany; 3 World Association of Zoos and Aquariums (WAZA), Gland, Switzerland; 4 UMR 5175, Centre d’Ecologie Fonctionnelle et Evolutive, campus CNRS, 1919 route de Mende, 34293, Montpellier Cedex 5, France; 5 Clinic for Zoo Animals, Exotic Pets and Wildlife, Vetsuisse Faculty, University of Zurich, Winterthurerstr. 260, 8057 Zurich, Switzerland

## Abstract

While it is commonly believed that animals live longer in zoos than in the wild, this assumption has rarely been tested. We compared four survival metrics (longevity, baseline mortality, onset of senescence and rate of senescence) between both sexes of free-ranging and zoo populations of more than 50 mammal species. We found that mammals from zoo populations generally lived longer than their wild counterparts (84% of species). The effect was most notable in species with a faster pace of life (i.e. a short life span, high reproductive rate and high mortality in the wild) because zoos evidently offer protection against a number of relevant conditions like predation, intraspecific competition and diseases. Species with a slower pace of life (i.e. a long life span, low reproduction rate and low mortality in the wild) benefit less from captivity in terms of longevity; in such species, there is probably less potential for a reduction in mortality. These findings provide a first general explanation about the different magnitude of zoo environment benefits among mammalian species, and thereby highlight the effort that is needed to improve captive conditions for slow-living species that are particularly susceptible to extinction in the wild.

Zoological gardens represent artificial environments in which animals are maintained, bred and displayed. By doing so, zoos achieve a diversity of goals beyond their visitors’ recreation: basic zoological and conservation education reaches 700 million visitors per year all over the world^1^. Continuing research and expertise building by many thousands of zoo staff worldwide continuously improves knowledge of animal, population and ecosystem management. Zoos also aim to maintain viable *ex situ* insurance populations of endangered species that can be used for re-introduction to the wild^2^,^3^. Zoo staff manages and generates funding for *in situ* conservation projects^1^,^4^. Finally, zoos facilitate opportunities for researchers to increase expertise in a large variety of areas, from basic zoology to applied husbandry and molecular biology.

When assessing the justification of holding nondomestic species in zoos, the welfare of the individual animals housed in captivity is a critical ethical issue that has to be weighed against these aims^5^. There is no single proxy to measure the welfare of animals. Indicators typically employed include measures of survival (such as longevity, annual survival, or ageing rate), reproduction (such as fertility or litter size), physiology (such as stress hormones or the occurrence of specific diseases) and behavior (such as stereotypies)^5^,^6^. It is typically believed that zoo animals live longer than their free-ranging conspecifics due to the consistent provision of food, water, and shelter from harsh climates, the absence of predation and management to minimize violent intraspecific encounters and accidents, as well as veterinary prophylactic and therapeutic intervention. However, zoo animals may be subject to behavioral deficits^6^. While an increasing number of comparative studies have demonstrated species-specific differences in the response to zoo-conditions^7^,^8^,^9^, and a few species-specific comparisons of survival metrics between free-ranging and captive specimens have been published^10^,^11^, large-scale inter-specific comparisons of captive and free-ranging populations have not yet been performed. Indeed, it is probably difficult to gather accurate demographic estimates in these two contrasted environments for a large range of species. In mammals, comparisons between wild and zoo populations published so far were based on a small number of species (consistently less than 25) and did not control for confounding effects of phylogeny^12^,^13^ or were restricted to a narrow taxonomic range (e.g. the mammalian order Artiodactyla^14^). In addition, these studies have led to conflicting results because they both failed^12^,^13^ and succeeded^14^ to detect the expected lower actuarial senescence rate (i.e. the rate of decrease in annual survival with increasing age) in zoos. Lastly, none of these studies included survival metrics other than senescence rate, such as longevity or age at the onset of senescence. Therefore, whether the common belief that mammals in zoos outlive their wild counterparts holds true remains unknown.

To address this question, we compared a set of survival metrics derived from life tables available from the literature for males and females of free-ranging populations of 59 mammalian species (including eight different orders, see Supplementary Figure S1) to those derived from the data on captive specimens of the same species from the Species360 database^13^,^15^ (formerly named International Species Information System database, ISIS). Based on these sex-specific life tables, four metrics describing the survival pattern of each species were calculated: longevity, baseline annual mortality, age at the onset of senescence and rate of senescence (Fig. 1). We compared these metrics between free-ranging and captive populations using linear models and controlled for phylogenetic relatedness among species using a mammal super-tree^16^. An expected higher longevity in zoos can originate from a lower baseline mortality, a later onset of senescence, a lower rate of senescence, or any combination of these measures (Fig. 1).

## Results

In 84% of species we analyzed (85% for males and 83% for females, including all carnivores), longevity was higher in zoos than in the wild for both sexes (Figs 2 and 3A). The positive relationship between longevity in the zoo and in the wild had a slope less than 1 (Table 1), indicating that short-lived species benefited from living in zoos to a higher extent than long-lived ones (Fig. 3A). In about 69% of the species (76% for males and 63% for females), the age at the onset of senescence was identical or delayed in zoos compared to the wild (Fig. 3B). The positive relationship between zoo and wild data of onset of senescence also had a slope less than 1 (Table 1), again indicating that species with an early onset of actuarial senescence delayed this onset in captivity to a larger extent than species with a late onset of actuarial senescence. For these latter species, often no difference in response to captivity occurred, and some species even displayed an earlier onset of senescence in zoos (Supplementary Figure S2). The slopes of the relationship between the baseline mortality (Fig. 3C) or the rates of actuarial senescence (Fig. 3D) at the zoo and in the wild were close to zero (Table 1), indicating that these metrics did not strongly covary between zoo and wild populations. While the baseline mortality was lower in zoos for about 62% of the species (61% for males and 64% for females) and the rate of senescence was lower in zoos for about 73% of the species (76% for males and 71% for females), the nearly horizontal slopes underline the importance of the species’ pace of life for these two metrics: species with a high baseline mortality and high rate of senescence in the wild (i.e. species with a faster pace of life) typically had lower values at the zoo. In contrast, species with a low baseline mortality and a low rate of senescence in the wild (i.e. species with a slower pace of life) typically had higher values at the zoo. Notably, mammals in zoos displayed less variation in both baseline mortality and rate of senescence than in the wild (Fig. 3), indicating more standardized conditions in zoos. All these patterns were remarkably similar for both sexes (Fig. 3).

## Discussion

Our findings indicate that, in general, a life in zoos allows mammals to live longer. However, our data suggest that the species-specific pace of life influences the extent to which a given species may benefit from captivity. Species with a faster pace of life typically suffer from high levels of environmentally-driven mortality in the wild including predation^17^, and zoos offer good protection against such causes of mortality. Mammals with a slower pace of life, however, are typically characterized by a later age at first reproduction, a longer gestation period, lower reproductive rates and lower annual mortality^18^. They do not benefit as much from living in zoos in terms of survivorship, or even have a slightly reduced longevity and higher senescence rates, which might be attributable to an earlier onset of breeding in these species in a zoo setting^19^,^20^. Thus, our broad-scale study supports previous work reporting that both Asian and African elephant females live longer in the wild than in zoos^10^ (Fig. 3) and provides a first general explanation why different species may benefit with different magnitudes from captivity. Data for the common hippopotamus^21^, included in Fig. 3 for a visual comparison alongside the elephant species, corroborate this interpretation. These findings emphasize that husbandry efforts to optimize the longevity of species with a slower pace of life should be intensified.

To what extent improvement of captive conditions has already occurred in zoos cannot be evaluated with our data. For long-lived species, we cannot include animals born in recent years because of the need to include only extinct cohorts to avoid overestimating age-specific mortality rates (i.e. only dead individuals can be included in life tables). If we assume that age-specific mortality decreases over time in zoos thanks to improved husbandry conditions, especially in recent years and independently of a species’ pace of life, then the absence of recent cohorts for long-lived species in our analyses might account at least partly for our finding that the survival benefit of living in zoos was less pronounced in long- than in short-lived species. We might expect improved living conditions in zoos to have delayed positive effects in long-lived species. For example, there has been tremendous effort in building new elephant enclosures in a great number of zoos in the last decade (DWHM and MC, pers. obs.) and large-scale studies have been performed on the potential to increase captive elephant welfare^22^. However, the benefits of such efforts on survival measures will not be detectable before many years from now. In this respect, it should be kept in mind that our findings, especially concerning the longer-lived species, mostly reflect past husbandry practices that are not necessarily representative any longer.

Our study refutes previous conclusions that the rate of actuarial senescence of vertebrates is not influenced by captivity^13^. When accounting for differences in the pace of life among species, we clearly demonstrate that faster-living species senesce at a lower rate in zoos than in the wild. In addition, we show that both males and females respond similarly to captive conditions. Such a discovery provides indirect evidence that the genuine sex differences in survival patterns in mammalian species subjected to high sexual selection^23^ involve physiological mechanisms and cannot only be explained by higher susceptibility of males to environmental conditions.

Carnivores show enhanced survival in zoos in our study, but are more susceptible to behavioral abnormalities^7^, highlighting the need for husbandry techniques to reduce these abnormalities while simultaneously maintaining the survival benefits. Although zoos offer simplified environments, social interactions might be as complex and challenging as in the wild, considering the high frequency of non-antagonistic contacts with humans and other species. Do animals, even when born and raised in zoos, perceive their enclosures as a spatial constraint in terms of compressed home ranges, or as an actual restriction of freedom in terms of a limitation of their own choices? Alternatively, do animals perceive zoos as a safe habitat where potential predators, food scarcity, or extreme climatic conditions are absent, allowing them to drastically reduce vigilance^24^? Our mere comparison of survival metrics between wild and captive populations should not be interpreted as a conclusive ethical judgment. Our findings should rather be considered as evidence that zoos generally enhance the longevity of mammals, except in species where there is little potential for such an enhancement because of their slower pace of life, which is already linked to both a low mortality and a high longevity in the wild. Because species with a slow pace of life are particularly threatened by extinction^25^, maintaining *ex situ* insurance populations of such threatened species remains a crucial conservation strategy.

## Methods

### Life tables

Zoo and wild population life tables were compiled from the Species360 database and literature, respectively (see Lemaître *et al.*^14^ for more details). Concerning free-ranging populations, publications containing life-tables from semi-captive populations were excluded to allow a strict comparison between captive and free-ranging populations. For 25 species, we collected several life tables from the same or different populations. When available, we gave preference to life tables obtained from longitudinal data. When several life tables of a given quality were available, we averaged them. When life tables were given in months or not with an integer of years, a standardization was made to obtain the survival at each integer age. For 9 species, the total number of individuals followed or considered was not given in the focal study. In such cases, we arbitrarily assumed that 100 individuals were considered per sex (close to the median value of the number of individuals alive at 1 year of age in the life tables we used). For wild life tables with a known total number of individuals (N = 50 species), the lowest was found for females of *Mustela vison* for which the life table only included 30 individuals, and the highest is observed for males of *Oryctolagus cuniculus* with a total of 9,020 individuals (Supplementary Table S1). For captive populations, we only used extinct cohorts of animals for which the sex and both birth and death dates were known, implying that animals were born in captivity. Extinct cohorts were defined as all cohorts born before a given year, which is determined as 2013 minus three quarters of the maximum longevity recorded for the species (Supplementary Table S1). As in captivity the sex ratio could be biased due to the culling of some young males during the first year for management issues (mostly in ruminants), we only computed parameters when at least 25 individuals for each sex of each species were alive at 1 year of age to get accurate estimates of age-specific survival. We finally obtained a dataset of 52 species for which data for both females and males were available, with one additional species with male-only data (leading to 53 species in males) and 6 additional species with female-only data (leading to 58 species in females) (Supplementary Table S1). For both captive and wild populations, we made the same calculations to obtain exactly comparable life tables. For visual comparison only, we included data from females of the two elephant species^26^,^27^ and sex-combined data from the common hippopotamus^21^ in the resulting data plots but did not include them in the analyses, as their data did not correspond to the data selection criteria stated above.

### Metrics of survival

**Figure 1:**
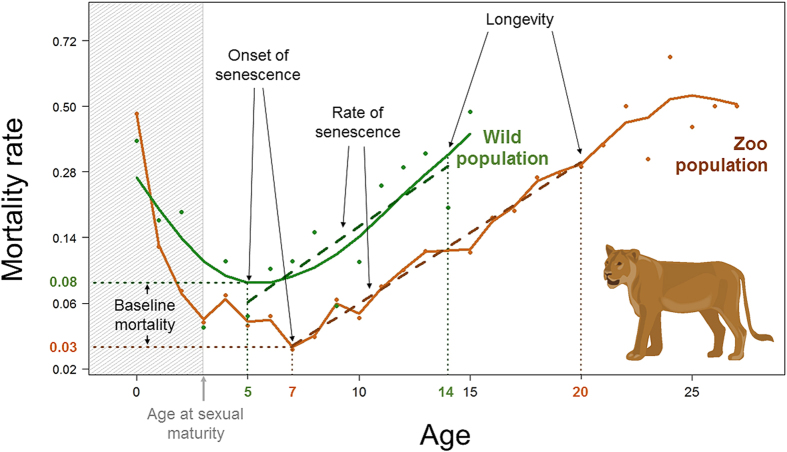
Graphical displays of the metrics of survival and actuarial senescence analyzed in this study. Data from female lions (*Panthera leo*) in zoo (in brown) and free-ranging (in green) conditions are used for illustrative purposes. Female lions in the zoo population live longer (age in years) and have a lower baseline annual mortality (in log%), a later onset of senescence (in years) and a lower rate of actuarial senescence (measured as the exponential rate of mortality increase per year).

**Figure 2:**
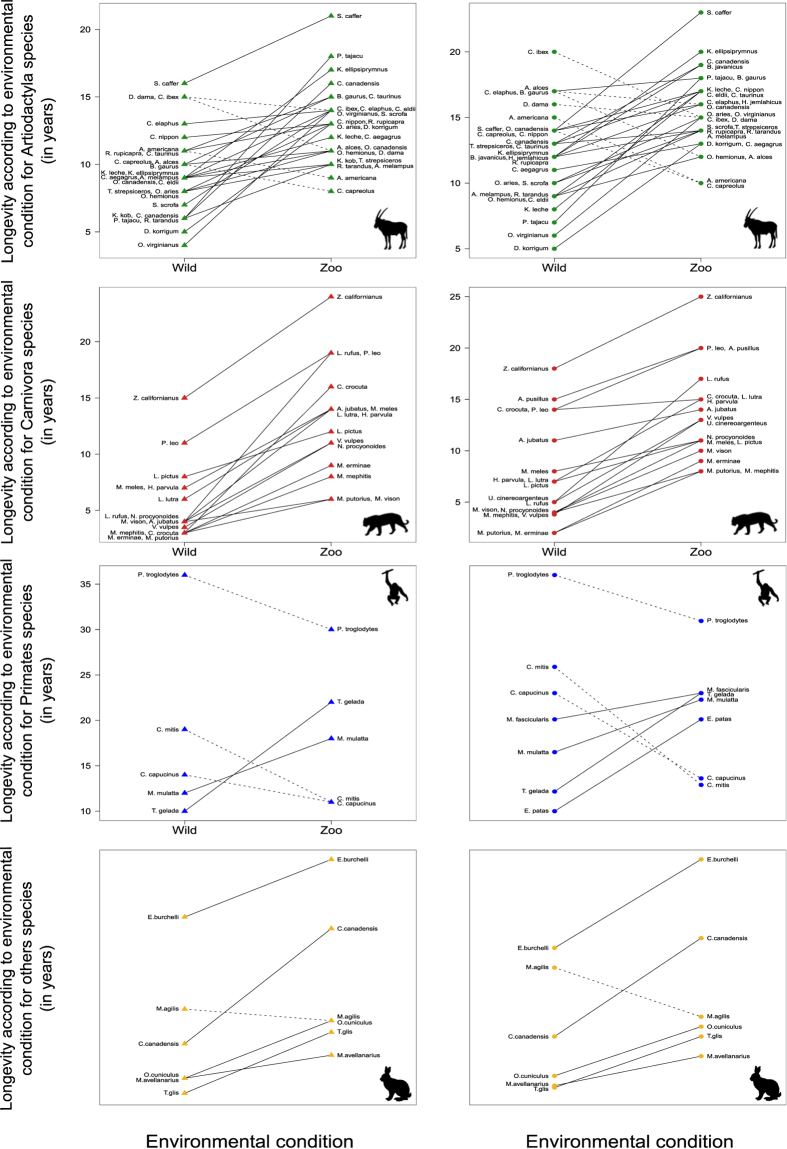
Longevity in free-ranging and zoo conditions for males (triangles) and females (circles) of each species of Artiodactyla, Carnivora, Primates and other orders (Diprotodontia, Lagomorpha, Perissodactyla, Rodentia and Scandentia). Species living longer in zoos are indicated with solid lines and species living shorter in zoos are indicated with dotted lines. Full species names are given in Supplementary Table S1. Animal pictures: nebojsa78©123RF.com.

**Figure 3:**
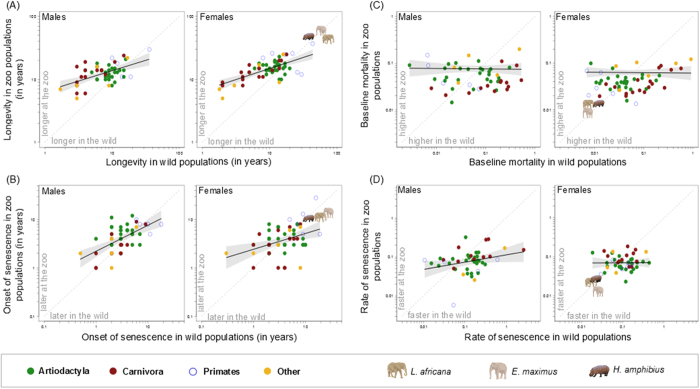
Comparison of (**A**) longevity, (**B**) age at the onset of senescence, (**C**) baseline annual mortality, and (**D**) rate of senescence (for males and females, respectively) between zoo and wild populations of 59 mammalian species. Points represent raw data, full lines represent the relationship between captive and wild estimates (on a log scale with 95% confidence interval of the model in grey) and the grey dashed line represents the equation y = x. For females, African (*Loxodonta africana*) and Asian (*Elephas maximus*) elephants and hippopotamus (*Hippopotamus amphibius*) were added for illustrative purposes, but were not included in the analysis.

To measure species- and sex-specific patterns of survival and actuarial senescence in captive and free-ranging populations, we used four distinct but complementary metrics: the longevity, the baseline annual mortality, the age at the onset of actuarial senescence and the rate of actuarial senescence (see Fig. 1. for a graphical display of these metrics). The longevity was extracted from species-specific life tables for both males and females and for both captive and wild populations (Supplementary Table S1). We defined longevity as the age at which 90% of individuals from the initial cohort (alive at 1 year of age) had died (Fig. 1). This allows avoiding spurious estimates due to the exceptionally long life of a few individuals^15^. However, this trait (called ‘longevity’ hereafter) is not a direct measure of senescence because it does not include any explicit information about age-dependent decline in survival. For other metrics, we first measured the logit-transformed age-specific mortality from a given life table. We thus constrained survival of 1 to be equal to 0.99, and we fitted a Generalized Additive Model to obtain the age-specific mortality curve. Since both theoretical and empirical evidence reveal that actuarial senescence does not start prior to the age of sexual maturity^28^,^29^, the onset of actuarial senescence was defined as the age at which the annual mortality rate was the lowest between the age at sexual maturity and the age at which 90% of individuals from the initial cohort have died (Fig. 1, Supplementary Table S1). The age at sexual maturity (in years) was collected for each sex and each species from a specific literature survey (Supplementary Table S2). The baseline mortality for each sex of each species and for both captive and free-ranging conditions was defined as the annual mortality observed at the age corresponding to the onset of senescence (Fig. 1, Supplementary Table S1). The baseline mortality at the onset of actuarial senescence corresponds to the lowest mortality observed for a given sex, species, and environment (i.e. wild or captive) between the age at sexual maturity and the age at which 90% of individuals from the initial cohort have died. The rate of senescence was measured as the slope of the linear regression of survival (on a log scale) on age computed between the age at the onset of senescence and the age at which 90% of individuals from the initial cohort have died (i.e. our measure of longevity)^30^ (Fig. 1, Supplementary Table S1). For short-lived species and life tables with a small sample size, we used the age at which at least 5 individuals of a given sex were still alive instead of the age at which 90% of the initial cohort was dead, both to achieve unbiased estimates due to the too few years lived by short-lived species, and to avoid estimating survival from less than 5 individuals. All estimates are reported in Supplementary Table S1 and displayed on Fig. 2 and Supplementary Figures S2–S4.

### Comparative analysis

To avoid biased assessment of the variation in survival patterns between captive and free-ranging populations, we controlled all the analyses for the non-independence between species due to shared ancestry using ‘Phylogenetic Generalized Least-Squares’ (PGLS) models^31^. A phylogeny was built for the 59 species (Supplementary Figure S1) using the phylogenetic super-tree of mammals published by Bininda-Emonds *et al.*^16^,^32^. Survival and senescence metrics were compared between free-ranging and captive males and females using linear models. Longevity and the onset and rate of actuarial senescence metrics were log-transformed, while baseline annual mortality was logit-transformed prior to any analysis. In a first part, the results of which are displayed in the main text, we analyzed the relationship between the metric measured in zoos (dependent variable) and the corresponding metric measured in wild populations (independent variable). A higher (for longevity and onset of senescence) or a lower (for baseline mortality and rate of senescence) value in captivity indicates that the focal species performs better in zoos. In a second part, we tested whether the quality of demographic estimates in the wild (i.e. measured from longitudinal or transversal studies) and species body mass (log-transformed) influenced the relationships between captive and wild metrics. Survival and senescence patterns are strongly associated with body mass^33^. Typically, larger species live longer^18^ and show a lower rate of senescence^34^ compared to small species, and have a slower pace of life^35^. To assess whether the patterns we report held when accounting for size differences among species, we included log-transformed body mass as a covariate in our models in a secondary analysis. We collected information about sex-specific mean adult body mass from the literature for each species analyzed (Supplementary Table S2). Therefore, for each of the four survival or senescence metrics in zoos and for a given sex, the full model included the corresponding wild metric and mean adult body mass as covariates, and the two-way interaction between the wild metric and data quality (as a fixed factor using longitudinal data as the reference). We then reduced the model by testing nested models by likelihood-ratio tests (LRT) so that the final model only included variables with statistically significant effects. A total of 3 nested models were tested for each of the metrics analyzed (Supplementary Table S3). A G-test was performed in each case. Models including the interaction between data quality and the wild estimates were never selected, whatever the survival or actuarial senescence metric considered (Supplementary Table S3). This suggests that quality of the wild demographic estimates did not influence the relationship between zoo and wild metrics. Moreover, we observed that body mass influenced zoo metrics in the same direction as the pace of life (Supplementary Table S4), leaving the patterns unchanged, whether including body mass in the models or not. All of these results are provided in Supplementary data (Supplementary Tables S3 and S4). For ruminant species, it has been shown that grazer species (whose natural diet consists mainly of grass) perform better than browser species (whose natural diet consists mainly of leaves or twigs) in captivity, in terms of survival and actuarial senescence^9^,^14^. In a complementary analysis, we therefore took this pattern into account and corrected the four survival and senescence metrics by including the percentage of grass in the natural diet of each ruminant species in the model. For all ruminant species, survival and senescence metrics were then adjusted for 60% (median, N = 27) of grass in the natural diet. However, results remained remarkably similar with or without this correction (Supplementary Table S5). All analyses were performed with R version 2.14.0^36^ and parameter estimates are given with the 95% confidence interval.

## Additional Information

**How to cite this article**: Tidière, M. *et al.* Comparative analyses of longevity and senescence reveal variable survival benefits of living in zoos across mammals. *Sci. Rep.* **6**, 36361; doi: 10.1038/srep36361 (2016).

**Publisher’s note:** Springer Nature remains neutral with regard to jurisdictional claims in published maps and institutional affiliations.

## Supplementary Material

Supplementary Information

## Figures and Tables

**Table 1:** Parameter estimates (with 95% confidence interval) of linear regressions (on the log-scale) linking the species-specific metrics obtained in zoo and free-ranging populations.

	Longevity		Baseline mortality		Onset of senescence		Rate of senescence	
	slope	intercept	slope	intercept	slope	intercept	slope	intercept
**Males**	53 species		51 species		51 species		45 species	
	**0.330** (0.191; 0.469)	**6.432** (4.461; 8.403)	**−0.010** (−0.053; 0.033)	**0.143** (0.113; 0.174)	**0.538** (0.330; 0.746)	**2.200** (1.122; 3.279)	**0.186** (0.001; 0.372)	**0.113** (0.092; 0.135)
**Females**	58 species		56 species		56 species		48 species	
	**0.351** (0.246; 0.456)	**6.621** (4.410; 8.833)	**−0.010** (−0.080; 0.061)	**0.115** (0.102; 0.128)	**0.327** (0.123; 0.532)	**2.413** (0.878; 3.947)	**0.005** (−0.178; 0.188)	**0.073** (0.055; 0.091)

Estimates were obtained from Linear Models when the phylogenetic signal λ was statistically not different from zero (i.e. for longevity, age at the onset of senescence and rate of senescence) and from Phylogenetic Generalized Least-Squares models when λ statistically differed from zero (for baseline mortality).
