# Supplementary material for: Comparative analyses of longevity and senescence reveal variable survival benefits of living in zoos across mammals
Source: Sci Rep. 2016 Nov 7;6:36361. doi: 10.1038/srep36361 (PMC5098244; doi:10.1038/srep36361)
Supplement: Supplementary Information [file srep36361-s1.pdf]

Supplementary Information

Comparative analyses of longevity and senescence reveal variable survival benefits of living in zoos across mammals

Morgane Tidière[1,*], Jean-Michel Gaillard[1], Vérane Berger[1], Dennis W.H. Müller[2], Laurie Bingaman Lackey[3], Olivier Gimenez[4], Marcus Clauss[5] and Jean-François Lemaître[1]

[1]Université de Lyon, F-69000, Lyon; Université Lyon 1; CNRS, UMR5558, Laboratoire de Biométrie et Biologie Evolutive, F-69622, Villeurbanne, France.

[2]Zoologischer Garten Halle GmbH, Fasanenstr. 5a, 06114 Halle (Saale), Germany.

[3]World Association of Zoos and Aquariums (WAZA), Gland, Switzerland.

[4]UMR 5175, Centre d'Ecologie Fonctionnelle et Evolutive, campus CNRS, 1919 route de Mende, 34293, Montpellier Cedex 5, France.

[5]Clinic for Zoo Animals, Exotic Pets and Wildlife, Vetsuisse Faculty, University of Zurich, Winterthurerstr. 260, 8057 Zurich, Switzerland.

*** Corresponding author:** mtidiere@gmail.com

Table S1: Survival and senescence data in males (M, N=53) and females (F, N=58) obtained from zoo and free-ranging populations of mammalian species. Data quality is indicated for free-ranging populations coded as factor with longitudinal (0) and transversal (1) data.

		SURVIVAL AND ACTUARIAL SENESCENCE DATA FROM ZOO POPULATIONS												SURVIVAL AND ACTUARIAL SENESCENCE DATA FROM FREE-RANGING POPULATIONS									
		Number of individuals alive at 1 year of age		First to last extinct cohorts included in the life table		Longevity (years)		Baseline mortality		Onset of senescence (years)		Rate of senescence		Data quality	Longevity (years)		Baseline mortality		Onset of senescence (years)		Rate of senescence		Nm, Nf* ; Wild life table references
**Species**	**Order**	**M**	**F**	**M**	**F**	**M**	**F**	**M**	**F**	**M**	**F**	**M**	**F**		**M**	**F**	**M**	**F**	**M**	**F**	**M**	**F**	
Acinonyx jubatus	Carnivora	739	725	1968-2000	1973-2000	14	14	0.042	0.036	2	2	0.103	0.107	0	4	11	0.400	0.100	2	2	0.407	0.044	124, 160; 1
Aepyceros melampus	Artiodactyla	310	593	1962-2001	1961-1999	10	14	0.171	0.059	7	4	0.104	0.090	1	9	9	0.038	0.045	2	2	0.200	0.168	334, 116; 2
Alces alces	Artiodactyla	101	132	1952-1994	1929-1997	11	12	0.102	0.087	5	3	0.090	0.042	0	10	17	0.129	0.047	2	9	0.052	0.163	288, 532; 3
Antilocapra americana	Artiodactyla	160	230	1911-1996	1911-2000	9	10	0.163	0.168	7	2	0.130	0.024	0	11	15	0.025	0.011	2	2	0.189	0.119	840, 840; 4
Arctocephalus pusillus	Carnivora	36	28	1964-1995	1962-1993	14	20	0.045	0.048	5	4	0.124	0.057	1	NA	15	NA	0.040	NA	6	NA	0.143	66; 5
Bos gaurus	Artiodactyla	110	121	1940-1999	1942-1994	15	18	0.043	0.046	7	6	0.082	0.062	1	10	17	0.031	0.050	3	6	0.203	0.067	72, 58; 6
Bos javanicus	Artiodactyla	96	113	1951-1999	1950-1994	14	19	0.071	0.049	4	3	0.058	0.041	1	NA	12	NA	0.050	NA	10	NA	NA	98; 7
Capra aegagrus	Artiodactyla	131	192	1966-2000	1966-1997	12	13	0.065	0.058	3	3	0.069	0.086	1	9	11	0.213	0.073	5	4	0.043	0.064	106, 159; 8
Capra ibex	Artiodactyla	183	290	1926-1997	1928-1997	14	15	0.074	0.059	6	5	0.098	0.058	0	15	20	0.012	0.018	4	4	0.174	0.043	215, 117; 9
Capreolus capreolus	Artiodactyla	75	101	1953-2003	1964-2003	8	10	0.188	0.131	6	3	0.318	0.058	0	10	14	0.121	0.036	4	3	0.106	0.089	829, 834; 10
Castor canadensis	Rodentia	82	79	1934-1994	1954-1995	16	17	0.092	0.089	4	6	0.025	0.055	1	6	7	0.280	0.211	2	5	NA	0.043	362, 249; 11
Cebus capucinus	Primates	31	33	1921-1997	1925-1992	11	13	0.246	0.115	8	5	0.086	0.065	0	14	26	0.008	0.009	10	19	0.615	0.165	98, 194; 12
Cercopithecus mitis	Primates	25	25	1964-1996	1967-1992	11	12	0.151	0.106	5	5	0.083	0.085	0	19	30	0.008	0.019	11	9	0.011	0.058	128, 58; 12
Cervus canadensis	Artiodactyla	299	478	1920-1997	1920-1992	16	19	0.079	0.042	5	4	0.049	0.056	1	6	13	0.297	0.091	3	9	0.060	0.219	**; 13–15
Cervus elaphus	Artiodactyla	230	382	1904-1997	1905-1993	14	16	0.081	0.042	6	4	0.080	0.078	0	13	17	0.023	0.031	5	3	0.221	0.095	767, 738; 16
Cervus eldii	Artiodactyla	272	316	1909-2000	1906-1998	14	17	0.071	0.043	3	8	0.082	0.128	0	9	9	0.043	0.191	2	2	0.109	0.056	47, 49; 17
Cervus nippon	Artiodactyla	550	774	1906-1995	1905-1994	13	17	0.132	0.062	6	3	0.032	0.042	0	12	14	0.021	0.015	4	4	0.148	0.161	149, 127; 18

Connochaetes taurinus	Artiodactyla	217	348	1925-1993	1933-1985	15	17	0.073	0.064	6	3	0.060	0.050	1	11	13	0.182	0.076	3	10	0.024	0.182	202, 211; 19
Crocuta crocuta	Carnivora	34	40	1920-1983	1930-1993	16	15	0.070	0.029	5	4	0.073	0.073	0	3	14	0.595	0.072	2	7	NA	0.077	97, 97, ; 20
Dama dama	Artiodactyla	516	828	1910-1994	1911-1991	11	15	0.153	0.068	3	3	0.038	0.052	0	15	16	0.003	0.019	7	9	0.146	0.095	25, 25; 21
Damaliscus korrigum	Artiodactyla	50	69	1967-1999	1966-1996	13	13	0.065	0.069	3	2	0.072	0.077	1	5	5	0.287	0.291	2	2	0.268	0.309	308, 188; 22
Equus burchellii	Perissodactyla	341	749	1901-1988	1900-1985	22	25	0.060	0.041	7	7	0.036	0.040	1	17	16	0.043	0.051	6	8	0.093	0.070	67, 61; 2
Erythrocebus patas	Primates	145	187	1949-1993	1948-1987	19	22	0.064	0.045	9	5	0.056	0.033	0	NA	8	NA	NA	NA	NA	NA	NA	120; 23
Helogale parvula	Carnivora	140	109	1963-1998	1963-2000	14	15	0.051	0.048	2	3	0.077	0.079	0	7	7	0.185	0.214	2	2	0.070	0.082	444, 402; 24
Hemitragus jemlahicus	Artiodactyla	278	380	1905-1993	1906-1994	14	16	0.097	0.063	6	4	0.077	0.055	1	NA	12	NA	0.056	NA	2	NA	0.089	55; 25
Kobus ellipsiprymnus	Artiodactyla	246	425	1939-1994	1938-1995	17	20	0.068	0.041	6	5	0.065	0.072	1	9	12	0.021	0.050	3	6	0.311	0.434	99, 100; 26
Kobus kob	Artiodactyla	40	77	1941-2000	1939-2000	10	15	0.042	0.031	4	4	0.131	0.098	1	6	NA	0.094	NA	1	NA	0.216	NA	340; 22
Kobus leche	Artiodactyla	202	328	1910-1993	1961-1991	12	17	0.126	0.047	4	7	0.039	0.073	1	9	8	0.072	0.071	3	3	0.210	0.224	**; 27
Lutra lutra	Carnivora	86	94	1961-1997	1971-2000	14	15	0.063	0.071	4	6	0.085	0.078	1	6	7	0.317	0.129	2	3	0.031	0.218	37, 36; 28
Lycaon pictus	Carnivora	480	437	1938-2002	1938-1994	12	11	0.045	0.057	3	3	0.176	0.144	0	8	7	0.135	0.247	3	2	0.072	0.036	101, 97; 29
Lynx rufus	Carnivora	64	42	1952-1985	1959-1987	19	17	0.022	0.040	6	1	0.076	0.046	1	4	5	NA	0.141	NA	4	NA	NA	80, 81; 30
Macaca fascicularis	Primates	129	136	1927-1991	1924-1985	21	26	0.060	0.030	16	13	0.145	0.057	0	NA	22	NA	0.010	NA	9	NA	0.130	184; 31
Macaca mulatta	Primates	63	159	1911-1991	1939-1985	18	25	0.054	0.033	8	8	0.075	0.050	0	12	17	0.077	0.036	7	7	0.033	0.098	44, 43; 32
Macropus agilis	Diprotodontia	157	182	1954-2001	1951-2001	8	9	0.243	0.176	2	1	0.026	0.053	1	9	14	0.074	0.123	2	8	0.173	NA	152, 152; 33
Meles meles	Carnivora	30	29	1972-1997	1972-1998	14	11	0.038	0.036	3	1	0.107	0.111	0	7	8	0.250	0.177	4	4	0.142	0.125	223, 307; 34,35
Mephitis mephitis	Carnivora	115	135	1908-2003	1908-2004	8	8	0.049	0.122	2	3	0.275	0.188	1	3	4	0.418	0.485	1	2	0.326	0.132	198, 181; 36
Muscardinus avellanarius	Rodentia	58	51	1990-2008	1988-2007	5	5	0.317	0.205	1	2	0.092	0.287	0	3	2	0.569	0.738	2	1	NA	NA	598, 470; 37
Mustela erminea	Carnivora	32	37	1973-2006	1973-2005	9	9	0.152	0.131	1	1	0.088	0.057	0	3	2	0.533	0.618	1	1	0.168	NA	75, 67; 38
Mustela putorius	Carnivora	45	44	1982-2008	1976-1997	6	8	0.067	0.136	1	3	0.284	0.191	1	3	2	0.323	0.324	1	1	0.373	NA	133, 65; 39
Mustela vison	Carnivora	25	28	1977-2006	1951-2004	6	10	0.186	0.150	2	3	0.204	0.073	1	4	4	0.333	0.349	3	2	NA	NA	36, 30; 40
Nyctereutes procyonoides	Carnivora	82	87	1948-2004	1950-2000	11	11	0.061	0.046	2	1	0.158	0.123	1	4	4	0.394	0.395	3	3	NA	NA	133, 136; 41,42
Odocoileus hemionus	Artiodactyla	66	103	1906-2002	1918-1998	11	12	0.122	0.094	5	6	0.083	0.130	0	8	9	0.146	0.161	5	2	0.159	0.019	**; 43
Odocoileus virginianus	Artiodactyla	206	330	1903-1990	1903-1990	14	15	0.137	0.090	11	3	0.069	0.040	1	4	6	0.433	0.329	2	1	0.196	0.037	**; 44
Oryctolagus cuniculus	Lagomorpha	293	354	1950-1998	1951-2005	8	8	0.166	0.168	2	3	0.092	0.138	1	3	3	NA	0.550	NA	3	NA	0.058	9020, 8306; 45
Ovis aries	Artiodactyla	420	471	1918-1996	1919-1986	13	15	0.110	0.074	5	2	0.029	0.049	0	8	10	0.198	0.109	7	4	NA	0.120	655, 667; 46,47

Ovis canadensis	Artiodactyla	229	247	1922-2000	1928-1998	11	16	0.130	0.080	3	3	0.049	0.057	0	9	14	0.133	0.071	3	4	0.115	0.083	404, 402; 48
Pan troglodytes	Primates	122	178	1921-1975	1926-1969	30	37	0.059	0.045	8	28	0.006	0.037	0	36	44	0.019	0.015	18	16	0.054	0.030	122, 144; 12
Panthera leo	Carnivora	966	1247	1900-1991	1900-1989	19	20	0.030	0.033	8	7	0.100	0.086	0	11	14	0.022	0.073	9	5	0.283	0.096	**; 49
Pecari tajacu	Artiodactyla	147	180	1910-1990	1935-1992	18	18	0.068	0.037	8	4	0.069	0.051	1	6	7	0.306	NA	4	NA	0.016	NA	**; 50
Rangifer tarandus	Artiodactyla	217	295	1924-2001	1924-1995	10	14	0.126	0.061	2	2	0.075	0.087	1	6	9	0.212	0.064	2	2	0.173	0.158	228, 160; 51,52
Rupicapra rupicapra	Artiodactyla	68	121	1969-2001	1914-2000	13	14	0.130	0.067	4	4	0.038	0.107	1	11	12	0.048	0.068	3	2	0.117	0.083	508, 359; 53
Sus scrofa	Artiodactyla	166	260	1940-2000	1943-1999	14	14	0.040	0.078	5	7	0.092	0.072	0	7	10	0.270	0.159	5	3	0.026	0.042	930, 853; 54
Syncerus caffer	Artiodactyla	120	174	1929-1992	1930-1991	21	23	0.020	0.022	12	8	0.111	0.074	1	16	14	0.030	0.075	5	7	0.120	0.138	172, 100; 2,22,55
Theropithecus gelada	Primates	74	101	1955-1991	1956-1989	22	26	0.043	0.019	10	10	0.044	0.072	1	10	11	0.123	0.119	6	5	0.208	0.136	301, 354; 56
Tragelaphus strepsiceros	Artiodactyla	391	660	1949-2000	1932-1992	10	14	0.095	0.078	2	3	0.097	0.069	0	8	13	0.197	0.028	3	6	0.128	0.167	**; 57
Tupaia glis	Scandentia	200	195	1965-2004	1965-2005	7	7	0.136	0.147	2	2	0.169	0.137	1	1.7	1.8	0.035	0.043	0.5	0.3	0.916	0.365	**; 58
Urocyon cinereoargenteus	Carnivora	25	27	1964-2005	1972-2001	6	13	0.133	0.066	2	4	0.315	0.125	1	NA	5	NA	0.228	NA	5.0	NA	0.181	**; 59
Vulpes vulpes	Carnivora	56	59	1945-1998	1954-2003	11	13	0.045	0.056	3	5	0.152	0.155	0	3.5	3.8	0.010	0.025	3.1	1.3	2.598	0.204	336, 249; 60
Zalophus californianus	Carnivora	178	217	1913-1990	1911-1967	24	25	0.034	0.029	9	8	0.051	0.057	0	15	18	0.075	0.018	6	8	0.055	0.164	96, 94; 61
Species included for visual comparison only:																							
Elephas maximus	Proboscidea		484		1960-2005	-	60	-	0.013	-	13	-	0.014	-	-	63	-	0.010	-	28	-	0.026	2905; 62†
Loxodonta africana	Proboscidea		302		1960-2005	-	45	-	0.019	-	12	-	0.025	-	-	71	-	0.009	-	21	-	0.023	1089; 63
Hippopotamus amphibius††	Artiodactyla	115		1961-1966		47		0.019		11		0.027		-	39		0.017		13		0.026		207; 64

* number of individuals included in life table for males and females respectively; ** the number of individuals included in life table is unknown and arbitrarily fixed to 100 per sex

† data from work-camp elephants ; †† data for both sexes combined (because data for free-ranging animals not separated by sex)

Table S2: Data on age at sexual maturity and mean sex-specific adult body mass (BM) of the mammalian species included in the analyses. Age at sexual maturity was obtained from De Magalhães and Costa (2009).

		**Age at sexual maturity (years)**		**Mean adult body mass (kg)**		**Reference for mean adult body mass**
**Species**	**Order**	**males**	**females**	**males**	**females**	
Acinonyx jubatus	Carnivora	1.25	1.25	42.3	37.9	65
Aepyceros melampus	Artiodactyla	1.08	1.25	56.9	43.8	66
Alces alces	Artiodactyla	1.68	2.06	241.45	187.16	67
Antilocapra americana	Artiodactyla	1.5	1.5	54	45.4	68
Arctocephalus pusillus	Carnivora	4.5	3.49	321	95	69
Bos gaurus	Artiodactyla	1.51	1.62	880	590	68
Bos javanicus	Artiodactyla	2	2	750	450	70
Capra aegagrus	Artiodactyla	1.88	1.11	33.8	20.125	68
Capra ibex	Artiodactyla	3.44	2.18	80.5	48.9	66
Capreolus capreolus	Artiodactyla	1.79	1.13	28	26.5	71
Castor canadensis	Rodentia	1.75	1.75	36.6	36.4	72
Cebus capucinus	Primates	8	4.12	3.23	2.28	12
Cercopithecus mitis	Primates	4.58	4.58	6.8	4.2	12
Cervus canadensis	Artiodactyla	2	2.33	312	238.667	73
Cervus elaphus	Artiodactyla	2	2.33	250	125	71
Cervus eldii	Artiodactyla	1.39	1.39	105	67	71
Cervus nippon	Artiodactyla	1.37	1.37	52	37	71
Connochaetes taurinus	Artiodactyla	2.5	1.13	235.3	184.9	66
Crocuta crocuta	Carnivora	1.89	3	80.2	73.6	72
Dama dama	Artiodactyla	1.39	1.33	67	44	71
Damaliscus korrigum	Artiodactyla	1.75	1.75	137	120.1	70
Equus burchellii	Perissodactyla	2.46	2.46	250	220	74
Erythrocebus patas	Primates	3.83	2.62	10	5.6	75
Helogale parvula	Carnivora	1.25	1.25	0.221	0.201	72
Hemitragus jemlahicus	Artiodactyla	2	1.5	103.3	56	66
Kobus ellipsiprymnus	Artiodactyla	2.11	2.11	231	174	75
Kobus kob	Artiodactyla	1	1.1	97.5	61.9	66
Kobus leche	Artiodactyla	2.87	2.46	104.3	78.7	66
Lutra lutra	Carnivora	1.5	1.5	10.3	7.4	76
Lycaon pictus	Carnivora	1.75	1.75	21.8	22.3	72
Lynx rufus	Carnivora	2	1	12	9	76
Macaca fascicularis	Primates	4.23	3.39	5.9	4.1	75
Macaca mulatta	Primates	5.49	3.37	6.2	3	75
Macropus agilis	Diprotodontia	1.16	1	19	11	75
Meles meles	Carnivora	1	1	11.6	10	76

Mephitis mephitis	Carnivora	0.92	0.92	2.6	2	76
Muscardinus avellanarius	Rodentia	0.92	0.92	0.033	0.033	76
Mustela erminea	Carnivora	1	0.26	0.321	0.213	76
Mustela putorius	Carnivora	0.88	0.85	0.987	0.623	76
Mustela vison	Carnivora	1.02	0.91	1.25	0.9	76
Nyctereutes procyonoides	Carnivora	0.83	0.83	3.47	3.8	77
Odocoileus hemionus	Artiodactyla	1.38	1.31	112.5	55.5	71
Odocoileus virginianus	Artiodactyla	1.14	0.85	154.5	68.5	71
Oryctolagus cuniculus	Lagomorpha	2	2	1.57	1.59	76
Ovis aries	Artiodactyla	2.5	1.5	26	20	75
Ovis canadensis	Artiodactyla	2.05	1.94	100	57.9	72
Pan troglodytes	Primates	7.99	9.24	39	31.3	12
Panthera leo	Carnivora	3	3	190	126	78
Pecari tajacu	Artiodactyla	0.98	0.9	20	22	72
Rangifer tarandus	Artiodactyla	1.86	1.81	106.5	76	71
Rupicapra rupicapra	Artiodactyla	1.83	1.85	40.3	31.7	66
Sus scrofa	Artiodactyla	0.19	0.91	90	70	76
Syncerus caffer	Artiodactyla	4.58	4.04	642.9	467.5	66
Theropithecus gelada	Primates	6	3.81	20.5	13.6	75
Tragelaphus strepsiceros	Artiodactyla	1.42	1.42	240.8	159.2	66
Tupaia glis	Scandentia	0.25	0.25	0.112	0.123	72
Urocyon cinereoargenteus	Carnivora	1	0.94	4.4	4.01	72
Vulpes vulpes	Carnivora	0.83	0.83	5.85	5.2	76
Zalophus californianus	Carnivora	5	3	392.5	110.6	76

References for Tables S1 and S2:

1. Kelly, M. J. *et al.* Demography of the Serengeti cheetah (*Acinonyx jubatus*) population: the first 25 years. *J. Zool.* **244,** 473–488 (1998).
2. Spinage, C. A. African ungulate life table. *Ecology* **53,** 645–652 (1972).
3. Ericsson, G. & Wallin, K. Age-specific moose (*Alces alces*) mortality in a predator-free environment: evidence for senescence in females. *Ecoscience* **8,** 157–163 (2001).
4. Byers, J. A. *American Pronghorn: Social Adaptations & the Ghosts of Predators Past*. (1997).
5. Gibbens, J. & Arnould, J. P. Age-specific growth, survival, and population dynamics of female Australian fur seals. *Can. J. Zool.* **87,** 902–911 (2009).
6. Ahrestani, F. S., Iyer, S., Heitkönig, I. M. A. & Prins, H. H. T. Life-history traits of gaur *Bos gaurus*: a first analysis. *Mammal Rev.* **41,** 75–84 (2011).
7. Choquent, D. Growth, body condition and demography of wild banteng (*Bos javanicus*) on Cobourg Peninsula, northern Australia. *J. Zool.* **231,** 533–542 (1993).
8. Magomedov, M. R. D., Akhmedov, E. G. & Nazrullaev, N. I. Demographic structure of the bezoar goat, *Carpa aegagrus* (Artiodactyla), population and regularities of its development in the eastern Caucasus. *Zool. Zhurnal* **83,** 234–240 (2004).
9. Toïgo, C. *et al.* Sex- and age-specific survival of the highly dimorphic Alpine ibex: evidence for a conservative life-history tactic. *J. Anim. Ecol.* **76,** 679–686 (2007).

10. Gaillard, J.-M., Viallefont, A., Loison, A. & Festa-Bianchet, M. Assessing senescence patterns in populations of large mammals. *Anim. Biodivers. Conserv.* **27,** 47–58 (2004).
11. Bergerud, A. T. & Miller, D. R. Population dynamics of Newfoundland beaver. *Can. J. Zool.* **55,** 1480–1492 (1977).
12. Bronikowski, A. M. *et al.* Aging in the natural world: comparative data reveal similar mortality patterns across primates. *Science* **331,** 1325–1328 (2011).
13. Knight, R. R. The sun river elk herd. *Wildl. Monogr.* 3–66 (1970).
14. Kimball, J. F. J. & Wolfe, M. L. Population analysis of a northern utah elk herd. *J. Wildl. Manag.* **38,** 161–174 (1974).
15. Houston, D. B. *The Northern Yellowstone Elk: Ecology and Management*. (1982).
16. Catchpole, E. A., Fan, Y., Morgan, B. J., Clutton-Brock, T. H. & Coulson, T. Sexual dimorphism, survival and dispersal in red deer. *J. Agric. Biol. Environ. Stat.* **9,** 1–26 (2004).
17. Nie, H., Song, Y., Zeng, Z. & Zhang, Q. Life history pattern and fitness of an endangered Hainan Eld's deer population. *Integr. Zool.* **6,** 63–70 (2011).
18. McCullough, D. R., Takatsuki, S. & Kaji, K. *Sika Deer: Biology and Management of Native and Introduced Populations*. (Springer Science & Business Media, 2008).
19. Attwell, C. A. M. Population ecology of the blue wildebeest *Connochaetes taurinus taurinus* in Zululand, South Africa. *Afr. J. Ecol.* **20,** 147–168 (1982).
20. Frank, L. G., Holekamp, K. E. & Smale, L. in *Serengeti II: Dynamics, Management, and Conservation of an Ecosystem* **2,** 364–384 (Sinclair A.R.E & Arcese P., 1995).
21. Saltz, D. Minimizing extinction probability due to demographic stochasticity in a reintroduced herd of Persian fallow deer *Dama dama mesopotamica*. *Biol. Conserv.* **75,** 27–33 (1996).
22. Mertens, H. Structures de population et tables de survie des buffles, topis et cobs de Buffon au Parc National des Virunga, Zaïre. *Rev. Décologie* **40,** 33–51 (1985).
23. Nakagawa, N., Ohsawa, H. & Muroyama, Y. Life-history parameters of a wild group of West African patas monkeys (*Erythrocebus patas patas*). *Primates* **44,** 281–290 (2003).
24. Waser, P. M., Elliott, L. F., Creel, N. M. & Creel, S. R. in *Serengeti II: Dynamics, Management, and Conservation of an Ecosystem* 421–447 (Sinclair A.R.E & Arcese P., 1995).
25. Caughley, G. Mortality patterns in mammals. *Ecology* **47,** 906–918 (1966).
26. Spinage, C. A. Population dynamics of the Uganda defassa waterbuck *(Kobus defassa ugandae neumann*) in the Queen Elizabeth Park, Uganda. *J. Anim. Ecol.* **39,** 51–78 (1970).
27. Sayer, J. A. & Lavieren, L. P. V. The ecology of the Kafue lechwe population of Zambia before the operation of hydro-electric dams on the Kafue River. *Afr. J. Ecol.* **13,** 9–37 (1975).
28. Kruuk, H., Conroy, J. W. H. & Moorhouse, A. Seasonal reproduction, mortality and food of otters *Lutra lutra L.* in Shetland. in **58,** 263–278 (1987).
29. Creel, S. R. & Creel, N. M. *The African Wild Dog: Behavior, Ecology, and Conservation*. (2002).
30. Crowe, D. M. Aspects of ageing, growth, and reproduction of Bobcats from Wyoming. *J. Mammal.* **56,** 177–198 (1975).
31. van Noordwijk, M. A. & van Schaik, C. P. The effects of dominance rank and group size on female lifetime reproductive success in Wild Long-tailed Macaques, *Macaca fascicularis*. *Primates* **40,** 105–130 (1999).
32. Meikle, D. B. & Vessey, S. H. Maternal dominance rank and lifetime survivorship of male and female rhesus monkeys. *Behav. Ecol. Sociobiol.* **22,** 379–383 (1988).

33. Stirrat, S. C. Age structure, mortality and breeding in a population of agile wallabies (*Macropus agilis*). *Aust. J. Zool.* **56,** 431–439 (2008).
34. Rogers, L. M., Cheeseman, C. L., Mallinson, P. J. & Clifton-Hadley, R. The demography of a high-density badger (*Meles meles*) population in the west of England. *J. Zool.* **242,** 705–728 (1997).
35. Wilkinson, D. *et al.* The effects of bovine tuberculosis (*Mycobacterium bovis*) on mortality in a badger (*Meles meles*) population in England. *J. Zool.* **250,** 389–395 (2000).
36. Casey, G. A. & Webster, W. A. Age and sex determination of striped skunks (*Mephitis mephitis*) from Ontario, Manitoba, and Quebec. *Can. J. Zool.* **53,** 223–226 (1975).
37. Juškaitis, R. Life tables for the common dormouse *Muscardinus avellanarius* in Lithuania. *Acta Theriol. (Warsz.)* **44,** 465–470 (1999).
38. Erlinge, S. Demography and dynamics of a Stoat *Mustela erminea* population in a diverse community of vertebrates. *J. Anim. Ecol.* **52,** 705 (1983).
39. Kristiansen, L. V., Sunde, P., Nachman, G. & Madsen, A. B. Mortality and reproductive patterns of wild European polecats *Mustela putorius* in Denmark. *Acta Theriol. (Warsz.)* **52,** 371–378 (2007).
40. Bonesi, L., Harrington, L. A., Maran, T., Sidorovich, V. E. & Macdonald, D. W. Demography of three populations of American mink *Mustela vison* in Europe. *Mammal Rev.* **36,** 98–106 (2006).
41. Helle, E. & Kauhala, K. Age structure, mortality, and sex ratio of the Raccoon dog in Finland. *J. Mammal.* **74,** 936–942 (1993).
42. Kowalczyk, R., Zalewski, A., Jedrzejewska, B., Ansorge, H. & Bunevich, A. N. Reproduction and mortality of invasive raccoon dogs (*Nyctereutes procyonoides*) in the Bialowieza Primeval Forest (eastern Poland). *Ann. Zool. Fenn.* **46,** 291–301 (2009).
43. Taber, R. D. & Dasmann, R. F. The dynamics of three natural populations of the deer *Odocoileus hemionus columbianus*. *Ecology* **38,** 233–246 (1957).
44. McCullough, D. R. *The George Reserve Deer Herd: Population Ecology of a K-Selected Species*. (Ann Arbor, 1979).
45. Gibb, J. A., White, A. J. & Ward, C. P. Population ecology of rabbits in the Wairarapa, New Zealand. *N. Z. J. Ecol.* 55–82 (1985).
46. Catchpole, E. A., Morgan, B. J. T., Freeman, S. N., Albon, S. D. & Coulson, T. N. *An integrated analysis of Soay sheep survival data*. (University of Kent, 1998).
47. Coulson, T. N. & Crawley, M. J. in *Soay Sheep: Dynamics and Selection in an Island Population* 328–331 (Clutton-Brock T.H. & Pemberton J.M., 2004).
48. Loison, A., Festa-Bianchet, M., Gaillard, J.-M., Jorgenson, J. T. & Jullien, J.-M. Age-specific survival in five populations of ungulates: evidence of senescence. *Ecology* **80,** 2539–2554 (1999).
49. Packer, C. *et al.* in *Reproductive Success* 363–383 (Clutton-Brock T.H., 1988).
50. Hellgren, E. C., Synatzske, D. R., Oldenburg, P. W. & Guthery, F. S. Demography of a collared peccary population in south texas. *J. Wildl. Manag.* **59,** 153–163 (1995).
51. Reimers, E. Mortality in Svalbard reindeer. *Ecography* **6,** 141–149 (1983).
52. Leader-Williams, N. *Reindeer on South Georgia*. (1988).
53. Bocci, A., Canavese, G. & Lovari, S. Even mortality patterns of the two sexes in a polygynous, near-monomorphic species: is there a flaw? *J. Zool.* **280,** 379–386 (2010).
54. Gamelon, M. *et al.* Do age-specific survival patterns of wild boar fit current evolutionary theories of senescence? *Evolution* **68,** 3636–3643 (2014).
55. Sinclair, A. R. E. *The African Buffalo: A Study of Resource Limitation of Populations*. (1977).

56. Dunbar, R. I. M. Demographic and life history variables of a population of Gelada baboons (*Theropithecus gelada*). *J. Anim. Ecol.* **49,** 485 (1980).
57. Owen-smith, N. Demography of a large herbivore, the Greater kudu *Tragelaphus strepsiceros*, in relation to rainfall. *J. Anim. Ecol.* **59,** 893–913 (1990).
58. Langham, N. P. E. The ecology of the Common tree shrew, *Tupaia glis* in peninsular Malaysia. *J. Zool.* **197,** 323–344 (1982).
59. Wigal, R. A. & Chapman, J. A. Age determination, reproduction, and mortality of the gray fox (*Urocyon cinereoargenteus*) in Maryland, U.S.A. *Z. Sauegetierkunde* 226–245 (1983).
60. Soulsbury, C. D., Baker, P. J., Iossa, G. & Harris, S. Fitness costs of dispersal in red foxes (*Vulpes vulpes*). *Behav. Ecol. Sociobiol.* **62,** 1289–1298 (2008).
61. Hernández-Camacho, C. J., Aurioles-Gamboa, D., Laake, J. & Gerber, L. R. Survival rates of the california sea lion, *Zalophus californianus*, in Mexico. *J. Mammal.* **89,** 1059–1066 (2008).
62. Mar, K. U. in *Giants on our Hands: Proceedings of the International Workshop on the Domesticated Asian Elephant* 195–211 (I. Baker, M. Kashio, 2002).
63. Moss, C. J. The demography of an African elephant (*Loxodonta africana*) population in Amboseli, Kenya. *J. Zool.* **255,** 145–156 (2001).
64. Laws, R. M. Dentition and ageing of the hippopotamus. *Afr. J. Ecol.* **6,** 19–52 (1968).
65. Krausman, P. R. & Morales, S. M. *Acinonyx jubatus*. *Mamm. Species* 1–6 (2005).
66. Bro-Jørgensen, J. The intensity of sexual selection predicts weapon size in male bovids. *Evolution* **61,** 1316–1326 (2007).
67. Sand, H. akan, Cederlund, G. & Danell, K. Geographical and latitudinal variation in growth patterns and adult body size of Swedish moose (*Alces alces*). *Oecologia* **102,** 433–442 (1995).
68. Loison, A., Gaillard, J.-M., Pélabon, C. & Yoccoz, N. G. What factors shape sexual size dimorphism in ungulates? *Evol. Ecol. Res.* **1,** 611–633 (1999).
69. Wickens, P. & York, A. E. Comparative population dynamics of fur seals. *Mar. Mammal Sci.* **13,** 241–292 (1997).
70. Bro-Jørgensen, J. Longevity in bovids is promoted by sociality, but reduced by sexual selection. *PLOS ONE* **7,** e45769 (2012).
71. Plard, F., Bonenfant, C. & Gaillard, J.-M. Revisiting the allometry of antlers among deer species: male-male sexual competition as a driver. *Oikos* **120,** 601–606 (2011).
72. Silva, M. & Downing, J. A. *CRC Handbook of Mammalian Body Masses*. (1995).
73. Mysterud, A. The relationship between ecological segregation and sexual body size dimorphism in large herbivores. *Oecologia* **124,** 40–54 (2000).
74. Ruckstuhl, K. E. & Neuhaus, P. Sexual segregation in ungulates: a comparative test of three hypotheses. *Biol. Rev.* **77,** 77–96 (2002).
75. Weckerly, F. W. Sexual-size dimorphism: influence of mass and mating systems in the most dimorphic mammals. *J. Mammal.* **79,** 33–52 (1998).
76. Carranza, J. Sexual selection for male body mass and the evolution of litter size in mammals. *Am. Nat.* **148,** 81–100 (1996).
77. Ward, O. G. & Wurster-Hill, D. H. *Nyctereutes procyonoides*. *Mamm. Species* **358,** 1–5 (1990).
78. Wilson, D. E. & Mittermeier, R. A. *Handbook of the Mammals of the World - Carnivores*. **1,** (Don E. Wilson, Russell A. Mittermeier, 2009).

Table S3: Model selection (model retained in bold) for each survival and actuarial senescence metric for the effect of data quality and body mass. P-values in bold indicate a statistically significant difference between competing models. A G-test was performed in each case.

		N species	Models compared	p-value
**Longevity**	Males	53	full model vs. model 2	0.221
			**model 2** vs. model 3	**0.015**
	Females	58	full model vs. model 2	0.104
			**model 2** vs. model 3	**0.003**
**Baseline mortality**	Males	51	full model vs. model 2	0.533
			**model 2** vs. model 3	**0.009**
	Females	56	full model vs. model 2	0.515
			**model 2** vs. model 3	**0.016**
**Onset of senescence**	Males	51	full model vs. model 2	0.602
			**model 2** vs. model 3	**0.002**
	Females	56	full model vs. model 2	0.482
			model 2 vs. **model 3**	0.091
**Rate of senescence**	Males	45	full model vs. model 2	0.728
			model 2 vs. **model 3**	0.199
	Females	48	full model vs. model 2	0.591
			model 2 vs. **model 3**	0.078

full model: Zoo ~ Wild + Body mass + Wild*Data quality
model 2: Zoo ~Wild + Body mass
model 3: Zoo ~Wild

Table S4: Parameter estimates from the selected model to assess the effect of data quality and body mass on the relationship between zoo and wild values of survival and actuarial senescence for 59 mammalian species.

	Sex	Method	Variables	β	95%CI	t	Adjusted-R^2	λ	95%CI
**Longevity**	Males	PGLS	Intercept	0.820	0.646;0.993	9.26	0.45	0.657	0.281;0.922
	53 species		Wild	0.140	-0.008;0.288	1.85			
			Body mass	0.109	0.064;0.153	4.78			
	Females	PGLS	Intercept	1.023	0.765;1.282	7.75	0.28	0.989	0.593;1
	58 species		Wild	-0.066	-0.196;0.064	-1.00			
			Body mass	0.123	0.072;0.174	4.72			
**Baseline mortality**	Males	PGLS	Intercept	-0.636	-1.207;-0.064	-2.18		> 0.999	0.405;1
	51 species		Wild	-0.001	-0.039;0.038	-0.03			
			Body mass	-0.201	-0.304;-0.098	-3.83			
	Females	PGLS	Intercept	-0.816	-1.273;-0.359	-3.50		0.992	0.760;1
	56 species		Wild	-0.028	-0.112;0.056	-0.65			
			Body mass	-0.167	-0.250;-0.083	-3.91			
**Onset of senescence**	Males	LM	Intercept	0.217	0.104;0.329	3.78	0.54	< 0.001	0;0.524
	51 species		Wild	0.390	0.207;0.574	4.17			
			Body mass	0.135	0.080;0.191	4.76			
	Females	LM	Intercept	0.489	0.196;0.782	3.27	0.14	0.325	0;0.754
	56 species		Wild	0.327	0.123;0.532	3.14			
**Rate of senescence**	Males	LM	Intercept	-0.946	-1.130;-0.762	-10.06	0.06	0.412	0;0.954
	45 species		Wild	0.186	0.001;0.372	1.97			
	Females	LM	Intercept	-1.137	-1.326;-0.949	-11.82	-0.02	0.483	0;0.872
	48 species		Wild	0.005	-0.178;0.188	0.06			

Table S5: Parameter estimates from the selected model to assess the relationship between zoo and wild values of survival and senescence for 59 mammalian species. Survival and actuarial senescence metric of captive ruminants are corrected for the percentage of grass in species' natural diet. Estimates obtained from models with or without the correction for captive ruminant species are similar.

	Sex	Method	Variables	β	95%CI	t	Adjusted-R^2	λ	95%CI
**Longevity**	Males	PGLS	Intercept	0.816	0.617;1.014	8.06	0.40	0.677	0.265;0.961
	53 species		Wild	0.138	-0.029;0.305	1.62			
			Body mass	0.113	0.062;0.163	4.39			
	Females	PGLS	Intercept	1.082	0.776;1.388	6.93	0.43	> 0.999	0.889;1
	58 species		Wild	-0.162	-0.279;-0.004	-2.70			
			Body mass	0.158	0.111;0.205	6.56			
**Baseline mortality**	Males	PGLS	Intercept	-0.833	-1.501;-0.165	-2.45		> 0.999	0.875;1
	51 species								
	Females	PGLS	Intercept	-0.928	-1.527;-0.329	-3.04		> 0.999	0.984;1
	56 species								
**Onset of senescence**	Males	LM	Intercept	0.217	0.105;0.329	3.79	0.54	< 0.001	0;0.531
	51 species		Wild	0.389	0.206;0.572	4.17			
			Body mass	0.135	0.080;0.191	4.78			
	Females	PGLS	Intercept	0.489	0.193;0.786	3.23		0.525	0.069;0.851
	56 species								
**Rate of senescence**	Males	LM	Intercept	-1.101	-1.190;-1.013	-24.30		0.565	0;0.983
	45 species								
	Females	PGLS	Intercept	-1.120	-1.232;-1.009	-19.73		0.373	0.006;0.794
	48 species								

Fig. S1. Phylogenetic tree built on the 59 mammalian species analyzed (based on Bininda-Emonds (2007, 2008)'s phylogenetic super-tree) according to species-specific mean adult body mass (in kg).

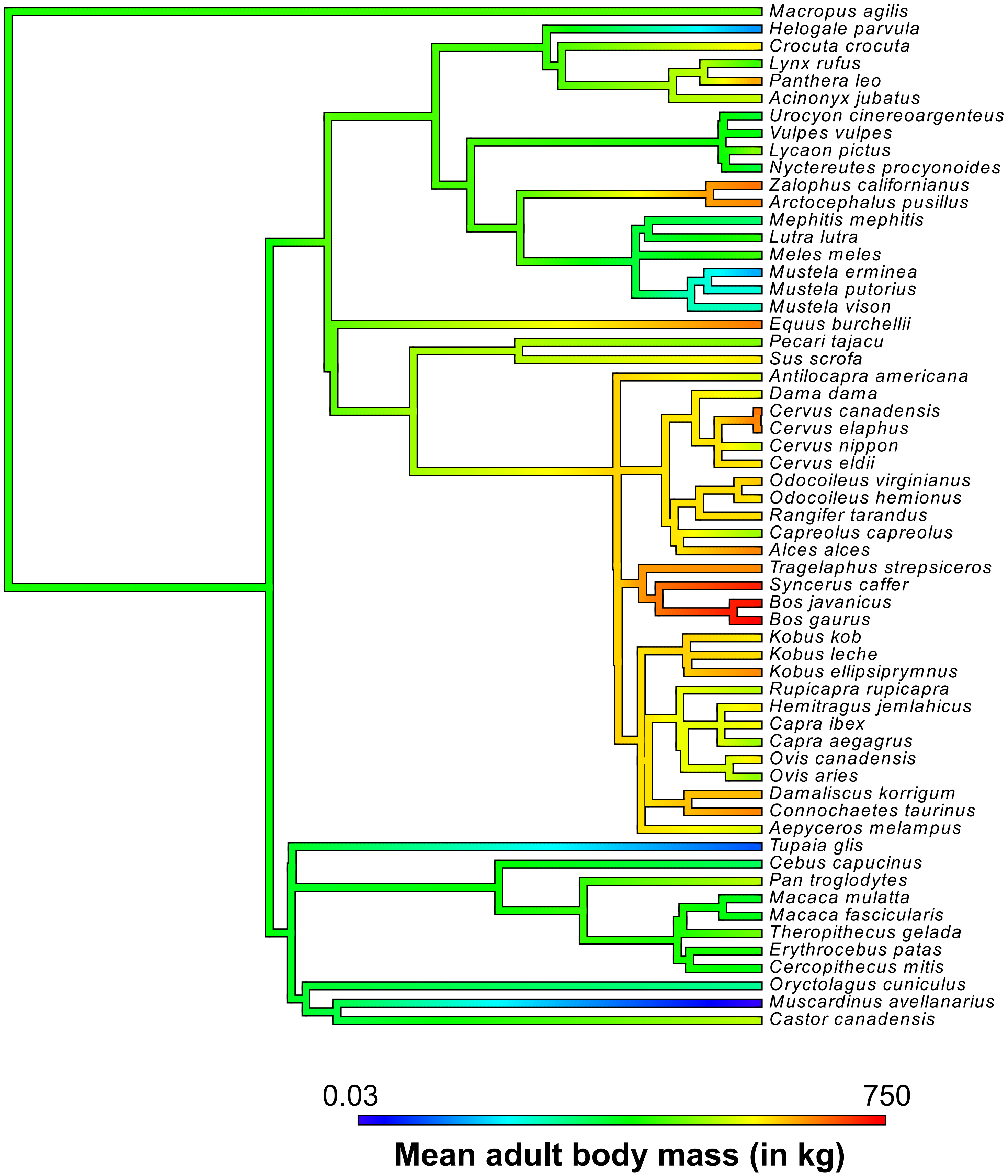

Figure S2. Onset of senescence in free-ranging and zoo conditions for males (triangles) and females (circles) of each species of Artiodactyla, Carnivora, Primates and other orders (Diprotodontia, Lagomorpha, Perissodactyla, Rodentia and Scandentia). Species senescing later in zoos are indicated with solid lines and species senescing earlier in zoos are indicated with dotted lines.

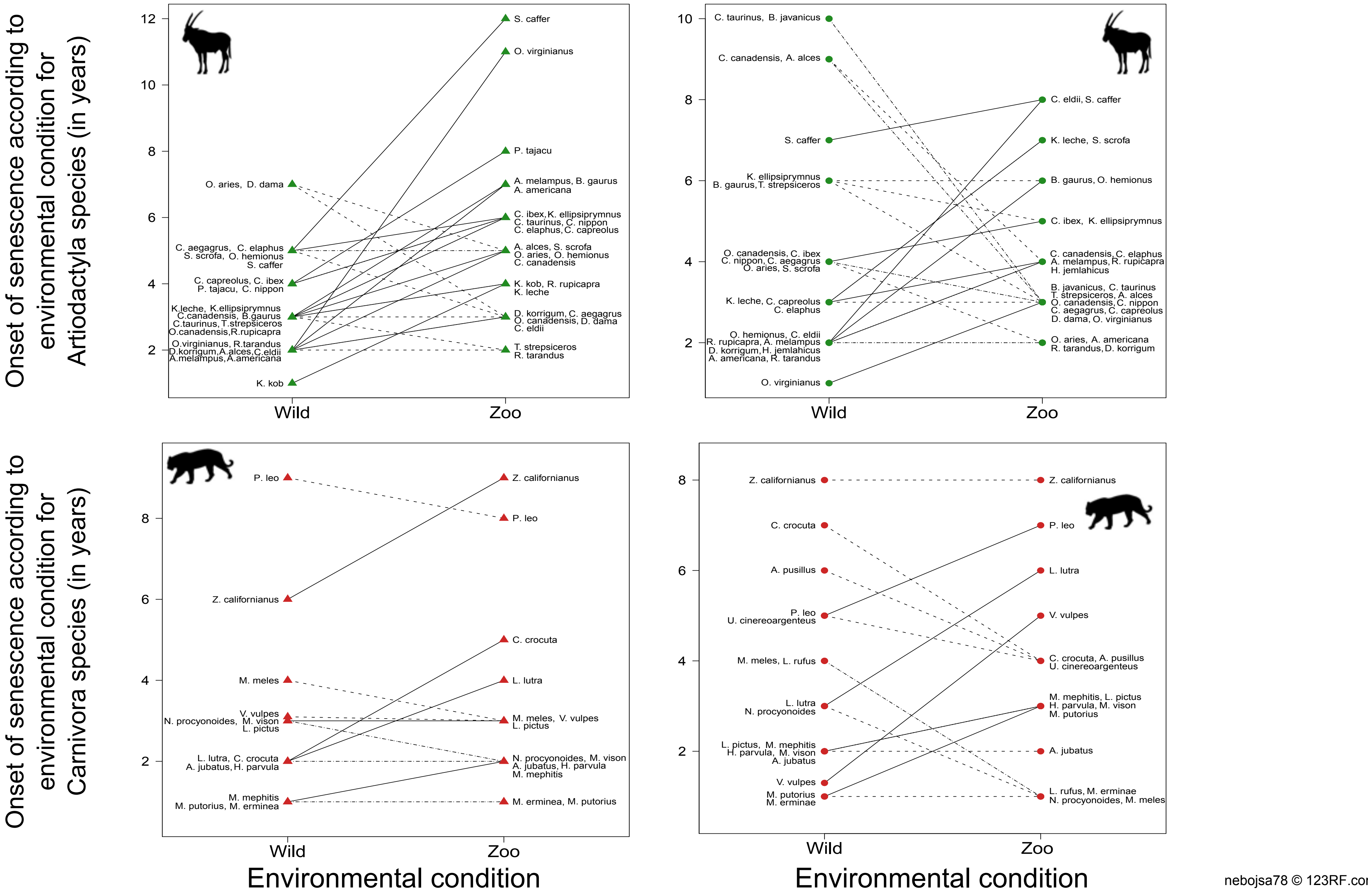

nebojsa78 © 123RF.com

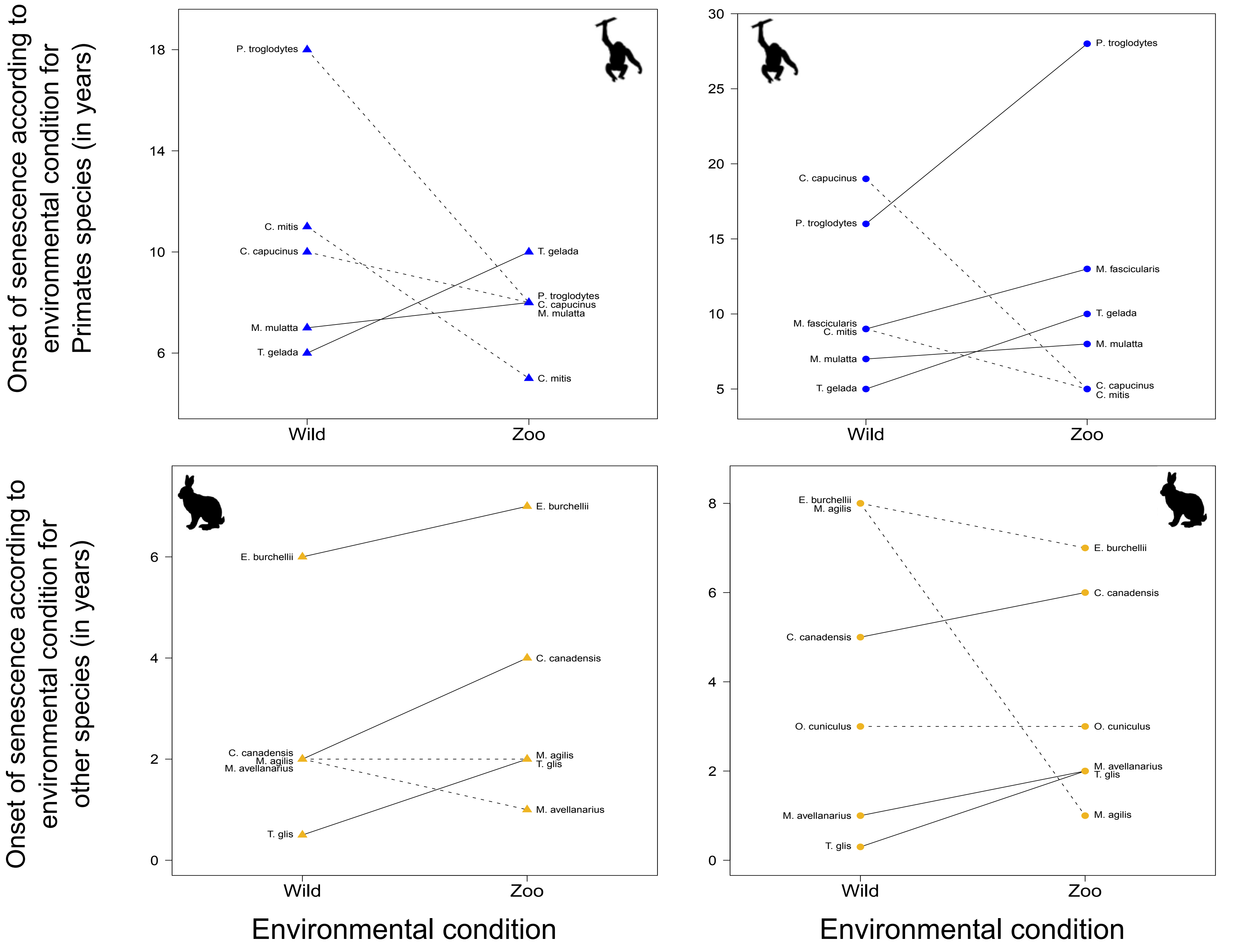

nebojsa78 © 123RF.com

Figure S3. Baseline annual mortality in free-ranging and zoo conditions for males (triangles) and females (circles) of each species of Artiodactyla, Carnivora, Primates and other orders (Diprotodontia, Lagomorpha, Perissodactyla, Rodentia and Scandentia). Species having a lower baseline mortality in zoos are indicated with solid lines and species having a higher baseline mortality in zoos are indicated with dotted lines.

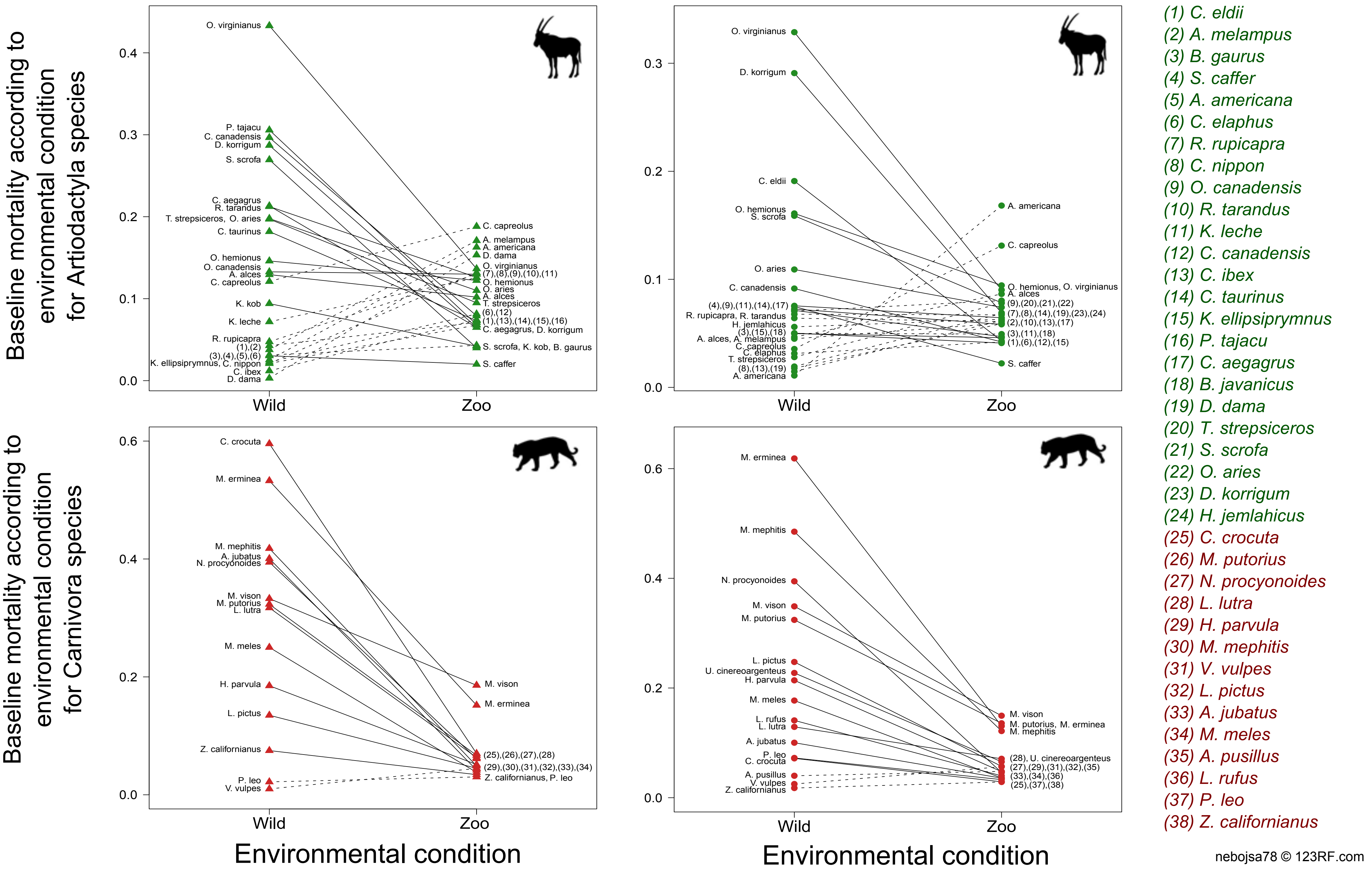

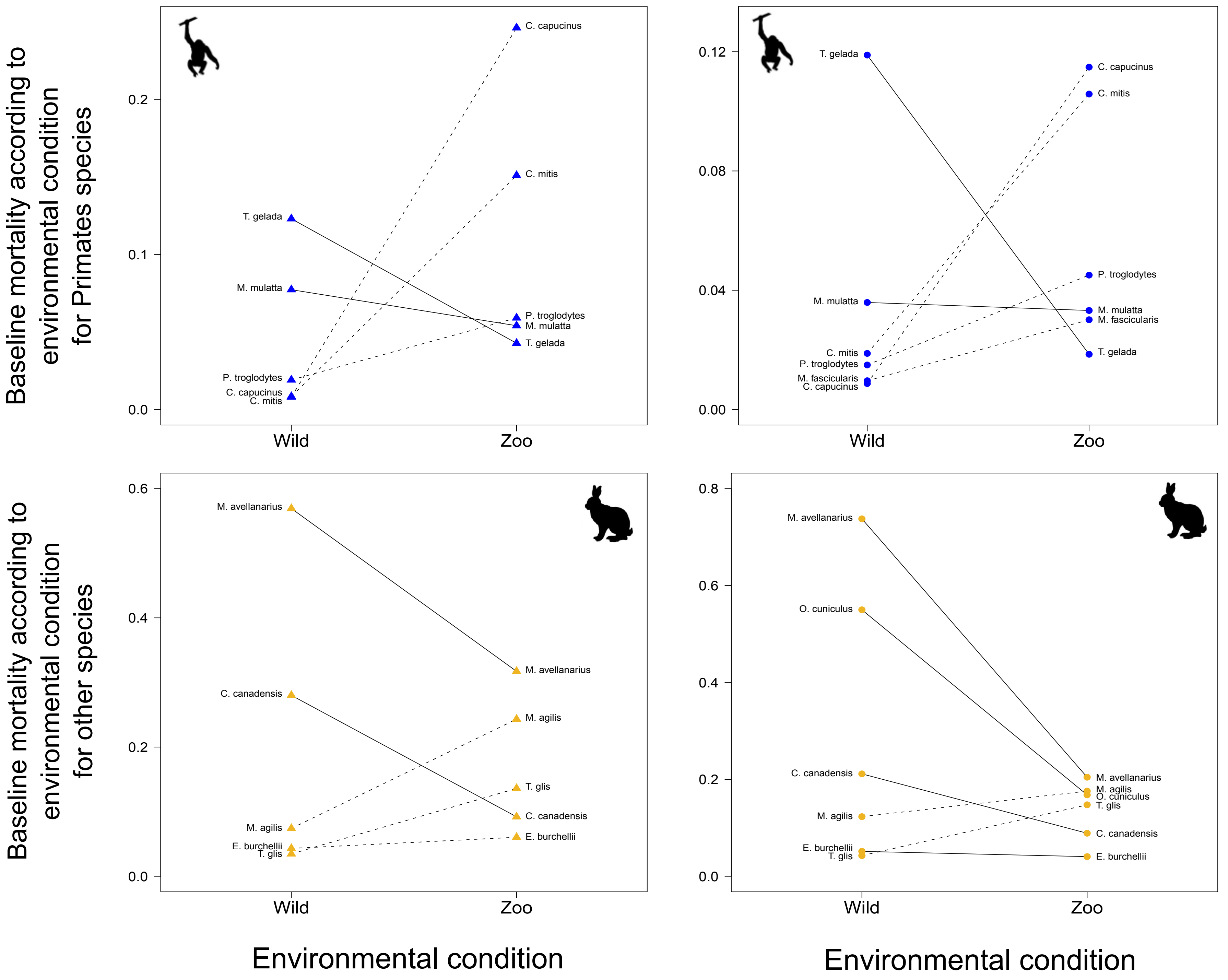

nebojsa78 © 123RF.com

Figure S4. Rate of senescence in free-ranging and zoo conditions for males (triangles) and females (circles) of each species of Artiodactyla, Carnivora, Primates and other orders (Diprotodontia, Lagomorpha, Perissodactyla, Rodentia and Scandentia). Species senescing slower in zoos are indicated with solid lines and species senescing faster in zoos are indicated with dotted lines.

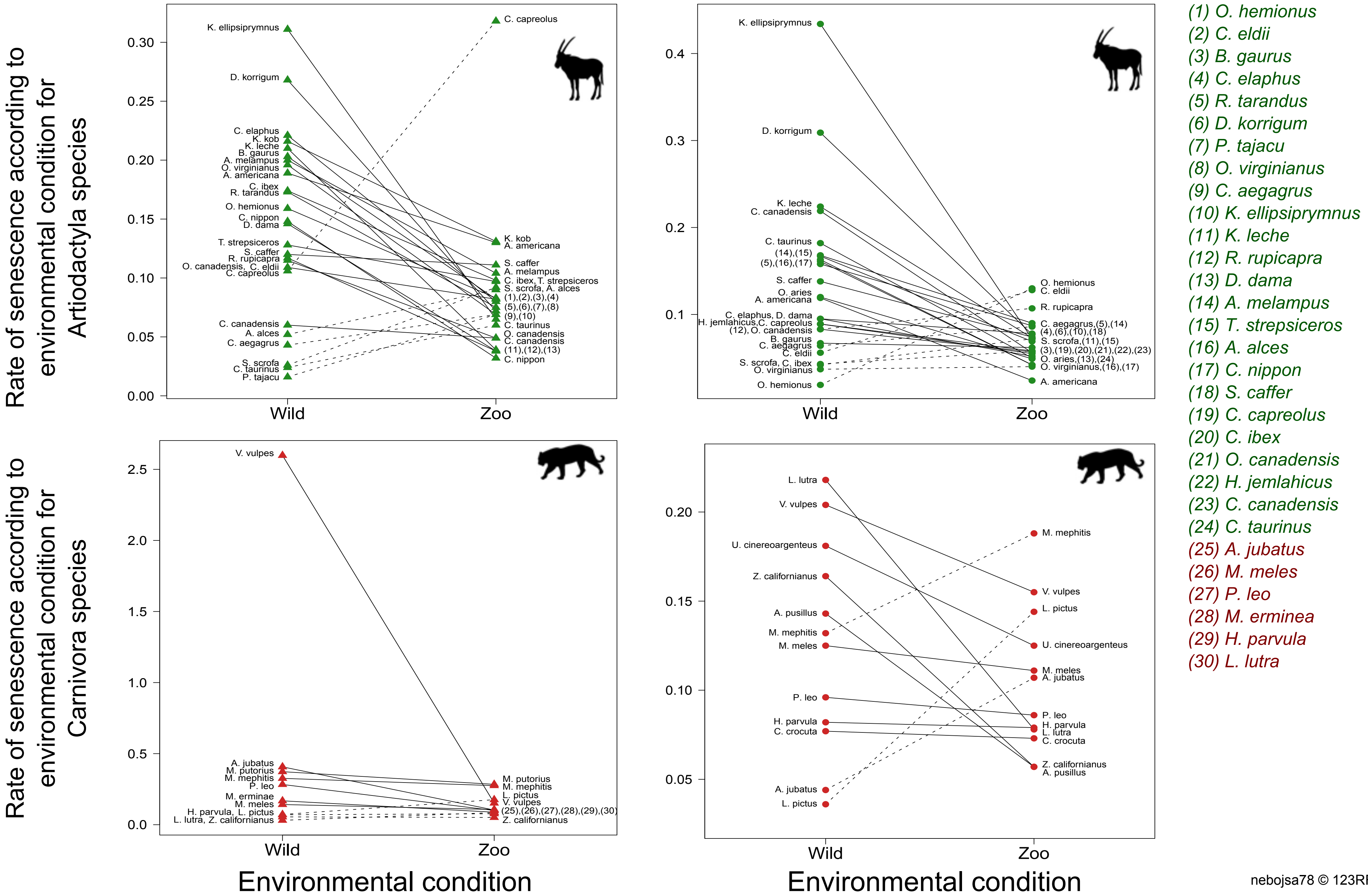

nebojsa78 © 123RF.com

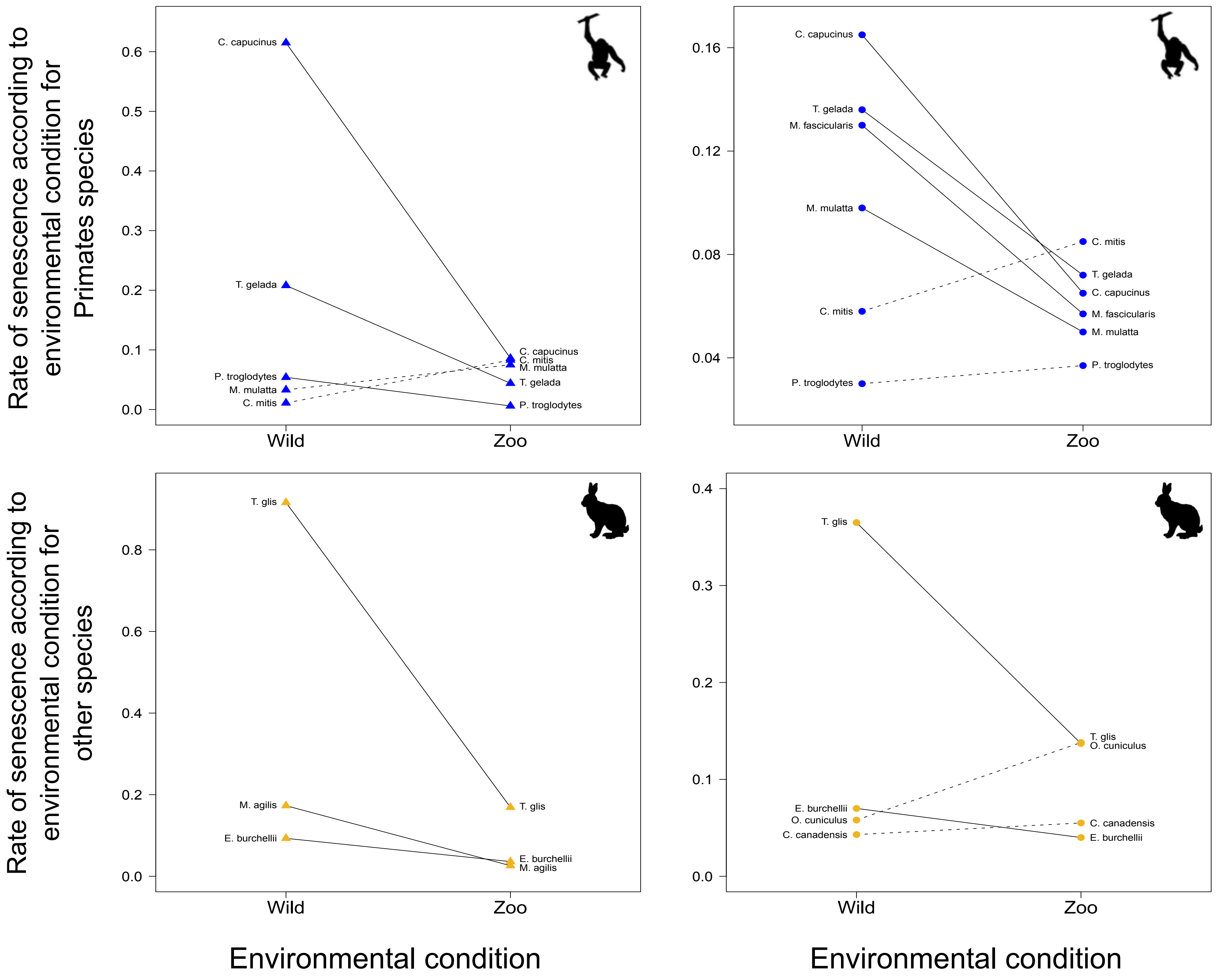

nebojsa78 © 123RF.com
